# Biopolymers for hydrogels in cosmetics: review

**DOI:** 10.1007/s10856-020-06390-w

**Published:** 2020-05-25

**Authors:** Stanisław Mitura, Alina Sionkowska, Amit Jaiswal

**Affiliations:** 1 President Stanisław Wojciechowski State University of Applied Sciences in Kalisz, Medical Faculty, Nowy Świat 4 st., 62-800 Kalisz, Poland; 2 Technical University of Liberec, Faculty of Mechanical Engineering, Department of Material Science, Liberec, Czech Republic; 3 Nicolaus Copernicus University in Torun, Faculty of Chemistry, Department of Chemistry of Biomaterials and Cosmetics, Gagarin 7 street, 87-100 Torun, Poland; 4 Centre for Biomaterials Cellular and Molecular Theranostics (CBCMT) VIT, Vellore, India

## Abstract

Hydrogels are cross-linked networks of macromolecular compounds characterized by high water absorption capacity. Such materials find a wide range of biomedical applications. Several polymeric hydrogels can also be used in cosmetics. Herein, the structure, properties and selected applications of hydrogels in cosmetics are discussed in general. Detailed examples from scientific literature are also shown. In this review paper, most common biopolymers used in cosmetics are presented in detail together with issues related to skin treatment and hair conditioning. Hydrogels based on collagen, chitosan, hyaluronic acid, and other polysaccharides have been characterized. New trends in the preparation of hydrogels based on biopolymer blends as well as bigels have been shown. Moreover, biopolymer hydrogels employment in encapsulation has been mentioned.

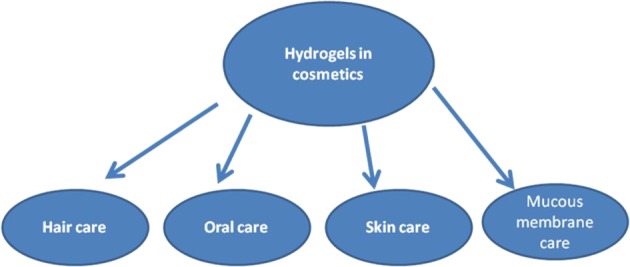

## Introduction

Hydrogels are three-dimensional polymeric networks held together by cross-linked covalent bonds and weak cohesive forces in the form of either hydrogen bonds or ionic bonds. This class of hydrophilic polymeric materials shows an inherent ability to swell in water and other suitable solvents and is capable of imbibing and retaining more than 10% of their weight in water within the gel structure. Hydrogels, together with other chemical compounds, can constitute a cosmetic formulation, usually called a cosmetic, which can find several topical applications on the body and hair surface. According to the EU and FDA regulations, a cosmetic is a substance or preparation intended for placement in contact with any external part of the human body, including the mucous membranes of the oral cavity and teeth. Functions cosmetics can perform are following: altering body odors, changing body appearance, cleansing skin and hair or maintaining them in good condition, perfuming or protecting [1, 2].

Skin is the largest organ of the body that is usually treated with cosmetic preparations. Its condition is essential since it provides a physical and chemical barrier and protects the body from various types of environmental influence. Skin contains numerous transducers continuously sending a wide range of sensory information to the brain for processing. From the cosmetic point of view, skin is responsible for external appearance, creating a unique and recognizable image identifiable to others. Skin condition depends on several factors. Structures of the stratum corneum, stratum spinosum and the function of sebaceous glands are also important. The sebaceous gland is responsible for sebum production. The largest sebaceous glands are distributed on the face, scalp, mid-back, and mid-chest, producing secretions released into the sebaceous duct which connects the gland to the follicular canal. Sebaceous secretions are produced in response to hormonal stimuli. Sebum has numerous functions on skin: it performs as a moisturizer, enhances barrier properties, and may act as an antifungal and antibacterial agent. When the skin barrier is damaged, reconditioning is required. For this purpose, a range of moisturizers which function temporarily until skin properties and barrier are reestablished can be used. There are three physiological mechanisms for rehydrating the stratum corneum, i.e. the use of occlusives, humectants, and hydrophilic matrices. Moisturizers assist in skin repair by creating environment suitable for regeneration. This may be accomplished with topically applied moisturizers by reducing the loss of water and creating a barrier on skin.

Hair, alike skin, is a complex biological system that performs specific functions. Hair consists principally of a protein called keratin and a small amount of lipids. Hair structure is affected by aging, lengthening and can also be influenced by environmental factors such as pollution and sunlight. When the hair structure is modified, some of natural properties of hair are changed as well. Cosmetic chemistry can support hair conditioning using appropriate chemical compounds to repair damaged hair.

In cosmetic preparation, several raw materials are used. Those employed in cosmetic formulations are selected to correspond with regulations which may vary and depend on a region and/or country. Formulating is a combination of art and science and relies on the knowledge of general, physical, inorganic and organic, polymer as well as raw materials chemistry. Numerous cosmetic ingredients are commercially available. Among them, synthetic polymers and biopolymers are extensively used. A polymer is a large molecule composed of repeating structural units typically connected by covalent chemical bonds. Natural polymers, also called biopolymers, are produced by living organism. A variety of natural polymers such as cellulose, the main component of wood and leaves, exists in nature. Another common biopolymer is starch widely used in food production. Some natural polymers are main components of skin and hair, e.g. collagen, elastin, keratin. These biopolymers, as well as other polysaccharides, are broadly applied in cosmetic formulations. Many natural polymers play a significant role in cosmetic formulation as moisturizers and thickening agents [3]. Based on biopolymers and synthetic polymers, a range of hydrogels can be formed for potential cosmetic and biomedical applications.

Hydrogels are cross-linked networks of the same or different types of synthetic polymers and/or biopolymers presenting high water absorption capacity. This high water absorption capacity is related to hydrophilic functional groups in the polymer structure such as amine (NH_2_), hydroxyl (-OH), amide (-CONH-, -CONH_2_) and sulfate (-SO_3_H) groups [4]. Due to the presence of hydrophilic functional groups, water molecules can migrate into the polymeric network which results in hydrogel expansion and occupation of larger volume. This process is called swelling. The amount of water absorption in different hydrogel types depends on the chemical structure of a synthetic polymer and/or biopolymer, cross-linking density and environmental conditions. Although in a polymer network the same water molecules are absorbed, water molecules can actually exist in three different states: free water, intermediate, and bound water molecules. Free water molecules are those which undergo the process of freezing at the freezing point, whereas no chemical bond exists between free water and the polymer functional groups. The amount of free water molecules usually depends on the hydrogel structure. In a compact hydrogel structure, the quantity of free water is lower. The second state of water or intermediate water can form weak interactions with functional groups in polymeric chains. Hydrogen bonding between polymeric chains and water molecules forms bound water and such water molecules are nonfreezing ones [5, 6].

**Scheme 1:**
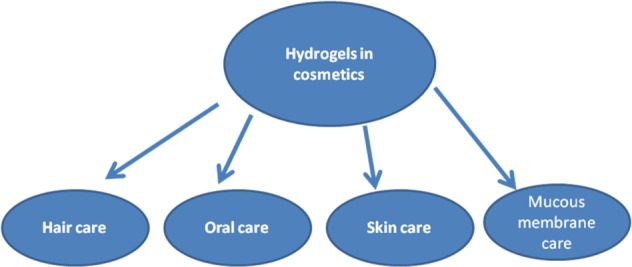
The common use of hydrogels in cosmetics

Hydrogels show high ability to swell in water or aqueous solutions. Due to a high amount of absorbed water these structures can be similar to human body tissues, as several human tissues contain indeed a large amount of water. This high ability to swell causes hydrogels to find an extensive scope of uses in various biomedical applications such as tissue engineering, regenerative medicine, and drug delivery. The hydrogel materials present certain advantages significant for biomedical applications. They can mimic the three-dimensional extracellular matrix environment in natural tissues. They can also be used in microcapsules and microparticles preparation for medical and cosmetic applications. In cosmetic applications, hydrogels are mainly applied topically, on skin, hair, and they are also used in oral care (Scheme 1). The use of bioadhesive hydrogels for skin care purposes presents important advantages, e.g. long residence times on the application site and a reduced product administration frequency. So far, several cosmetic formulation have been prepared as hydrogels containing active cosmetic ingredients. The selected hydrogels are suitable bioadhesive hydrogel formulations for cosmetic application on skin. Hydrogels used in cosmetic preparations can be based on numerous biopolymers, i.a. collagen, gelatin, hyaluronic acid, alginate, chitosan, xantan gum, pectin, starch, cellulose and its derivatives. Biopolymer-based hydrogels are used for developing new cosmetic products, such as so-called “beauty masks”. These masks are claimed to hydrate skin, restore its elasticity and promote anti-aging performance. Superabsorbent hydrogels, particularly acrylate-based materials, are extensively applied in hygiene products to absorb fluids as they are able to keep moisture away from skin, promoting skin health, preventing diaper rash and providing comfort.

Synthetic polymers such as poly (vinyl alcohol), polyacrylamide, poly (ethylene oxide) and poly (ethylene glycol) have been used for hydrogel formation. These hydrogels are mainly used in biomedical applications. Natural polymers usually present higher biocompatibility when compared to synthetic polymers. But, on the other hand, synthetic ones are chemically stronger than natural ones [7, 8]. Moreover, the reproducibility of the final microstructures and properties of biopolymer-based hydrogels is difficult to control between experiments, so hydrogels composition may vary from one batch to another. In contrast, synthetic hydrogels are more reproducible, although their final structure can depend on polymerization conditions and environment control.

## Collagen in cosmetics

**Fig. 1:**
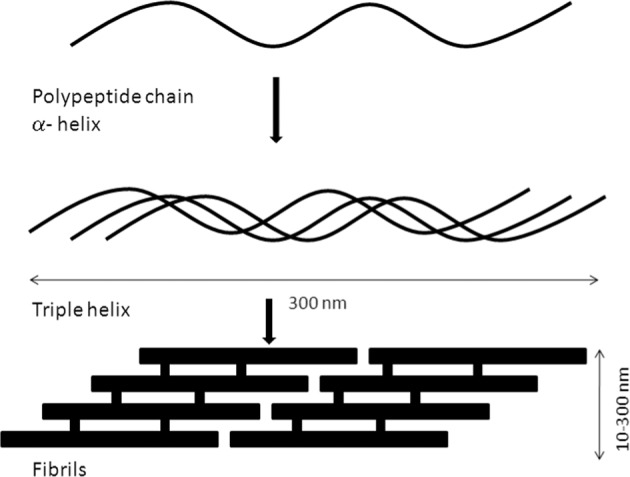
Hierarchical structure of collagen

Collagen is a structural protein in animals where it provides fundamental structural and mechanical support [9–11]. Collagen is not the only one simple protein. The collagen family encompasses 29 genetically distinct collagen types. The major collagen types involve type I (found in skin, tendon and bone tissues), type II (cartilage), and type III (skin and vasculature). These collagen types constitute parts of fibrillar structures responsible for tissue architecture and integrity. In cosmetic applications, type I collagen is prevalently used. Each chain of type I collagen contains 1000 amino acids and forms an α-helix. The sturdy structure is shaped by a repeated sequence of three amino acids. The hierarchical structure of type I collagen is shown in Fig. 1. An amino acid analysis of collagen revealed that every third amino acid is glycine, a small amino acid that fits perfectly into the helix. Many of the remaining positions in the chain are filled with two unexpected amino acids, i.e. proline and a modified version of proline: hydroxyproline. The latter, responsible for collagen stability, is obtained by modifying regular proline amino acids after the collagen chain has been built. Within a fibril, collagen is stabilized by numerous intra- and intermolecular forces. In the collagen triple helix stabilization, the main role is fulfilled by hydrogen bonds. The alignment of charged groups between collagen molecules contributes to electrostatic interactions and is important in defining the intra-molecular structure. Each collagen molecule is strongly molecularly connected with neighboring collagen molecules and an applied force can be transmitted through a fibril to each collagen molecule. Collagen is a highly cross-linked material usually insoluble in both water and oils. Since insoluble in water, it is rather not a raw material appropriate for cosmetic preparation. Only collagen obtained from a very young organism is soluble in aqueous solutions. In a solution, it is very sensitive to UV irradiation and high temperature [12].

Collagen can be obtained from several animal tissues. Bovine collagen has been widely applied; however, it is burdened with the risk of bovine spongiform encephalopathy (BSE) and transmissible spongiform encephalopathy (TSE) development. Scientists are looking for safer alternatives, for example collagen extracted from marine sources (fish skin and scales, marine sponges or jellyfish umbrella). In the case of cosmetic creams containing collagen obtained from the marine sponge and creams containing mammal collagen, when comparing their impact on skin, it can be noticed that their effects on the skin pH, moisture and sebum are comparable. This means that animal collagen can be successfully replaced with marine sponge collagen for cosmetic preparations. Collagen can be produced through fermentation in the presence of bacteria, for example *Methylobacterium*, *Solibacter usitatus* and *Rhodopseudomonas palustris*. Notably, the collagen source affects its properties, for example marine collagen is characterized by stability lower than that of chicken collagen.

In a collagen molecule, atoms in individual chains are held together with covalent bonds, while the three chains are held in a triple-helical structure by weaker bonds. These week bonds are hydrogen, dipole-dipole, and ionic bonds as well as van der Waals interactions. When the protein is heat-denatured, these weak bonds are broken, but the covalent bonds stay intact and the three chains separate from one another and collapse into random coils. Collagen, like most proteins, loses all of its structure upon heating. The triple helix unwinds and the chains separate. This denatured mass of the tangled chains is called gelatin. Gelatin itself is a mixture of water-soluble proteins derived primarily from collagen. Gelatin usually binds more water than collagen as it is partially degraded collagen, and thus, more active groups are exposed to interactions with water via hydrogen bonds. Based on their structural roles and compatibility within the body, collagen and gelatin are commonly used biomaterials in medical, pharmaceutical and cosmetic industries. The thermal denaturation temperature (so called melting temperature) of collagen depends on water content, the environmental medium pH, and cross-linking degree [12]. Both, collagen and gelatin, can be used for hydrogels preparation.

In cosmetic formulation, due to collagen insolubility, mainly hydrolyzed collagen is used. Small peptides and short polypeptides are well-soluble in water and can be easily incorporated into several cosmetic formulations [13]. However, if the molecular weight of hydrolyzed collagen peptides is too small, it is not easy to prepare good quality hydrogels.

Hydrogels made of collagen are usually prepared by the freeze-drying technique. Self-aggregation of water-dispersible collagen under freeze-drying conditions is appropriate for creating collagen hydrogels and aerogels for pharmaceuticals and cosmetics uses [14]. Hydrogels can be made not only of pure collagen but also of the blend of collagen with other hydrophilic synthetic polymers and/or biopolymers [12]. Collagen was used for preparing composites made of collagen/gelatin/hydroxyethyl cellulose characterized by high swelling properties [15]. Collagen hydrogels were proposed for 3D multicellular microfluidic chip construction applied in an *in vitro* skin model which can function as a more physiologically realistic platform for testing skin reactions to cosmetic products and drugs [16]. Collagen-based hydrogels can be cross-linked with EDC/NHS, starch dialdehyde, tannic acid, squaric acid, PEG-dialdehyde UV radiation and many other cross-linking agents [17–21].

Gelatin is a denatured form of collagen obtained by acid or alkaline collagen processing. Gelatin has proven to be a cheaper collagen alternative which forms a hydrogel responding to change in temperature values. Pure gelatin and gelatin-based hydrogels have been extensively studied for several biomedical and pharmaceutical applications. For photosensitive gelatin-based hydrogels preparation, manufacturing technologies such as ultraviolet stereolithography and two-photon polymerization have been employed [22].

Collagen-based hydrogels are widely used in reconstructive medicine and pharmacy. Properties of collagen-based materials are influenced by a collagen source as well as a preparation method involving purification, fibril formation, or casting and subsequent cross-linking. Nowadays, for cosmetic applications, mainly collagen derived from fish skin is considered. Bovine collagen and pig collagen can be dangerous due to several animal-derived diseases. The main problem fish collagen poses is its low denaturation temperature, much lower than the human body temperature. The best way to apply collagen in cosmetics is to hydrolyze it to polypeptides of small molecular weight which are able to penetrate into the skin.

One of the most commercially thriving collagen uses is the subcutaneous injection of soluble collagen for repairing dermatological defects [13]. In addition to wrinkles reduction, nasolabial folds correction and acne scars healing were observed. Collagen fillers differ depending on types of substances which, in addition to collagen, are added to a given formulation. They are introduced in order to obtain a more permanent effect, increasing the viscosity (this allows the filler to correct the deeper defects). The study of collagen for cosmetic applications is carried out by several research groups around the world.

## Chitosan in cosmetics

**Fig. 2:**
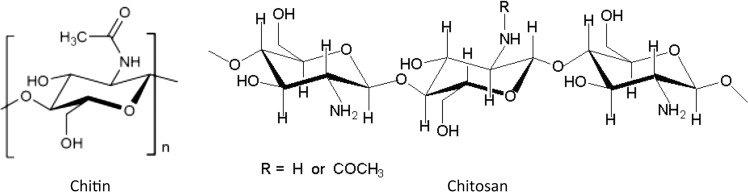
Structure of chitin and chitosan [12]

Chitosan is a natural cationic polyelectrolyte copolymer derived from chitin. Chitin is a homopolymer comprised of 2-acetamido-2-deoxy-β-D-glucopyranose units. A majority of units in chitosan chains exists in the deacetylated form as 2-amino-2-deoxy-β-D-glucopyranose. When chitin is deacetylated to at least 50%, it becomes soluble in dilute acids and is referred to as chitosan. Chitin and chitosan structures are shown in Fig. 2.

Chitosan is a biodegradable natural polymer presenting great potential for pharmaceutical and cosmetic applications due to its biocompatibility, high charge density, non-toxicity and mucoadhesion. Recently, chitosan has attracted attention because the range of its applications has been extended to medical, waste water treatment, biomembranes, hydrogel development and cosmetic formulations. In cosmetic formulation, chitosan is used in the production of mascaras, hair conditioners, hair foams, body creams [23, 24].

**Fig. 3:**
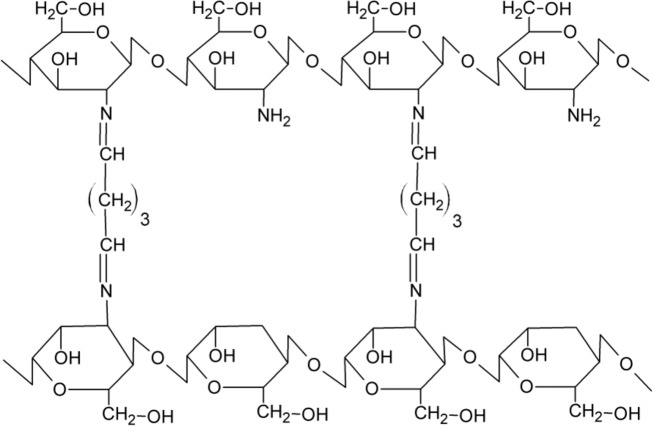
Example of chemically cross-linked chitosan [12]

**Fig. 4:**
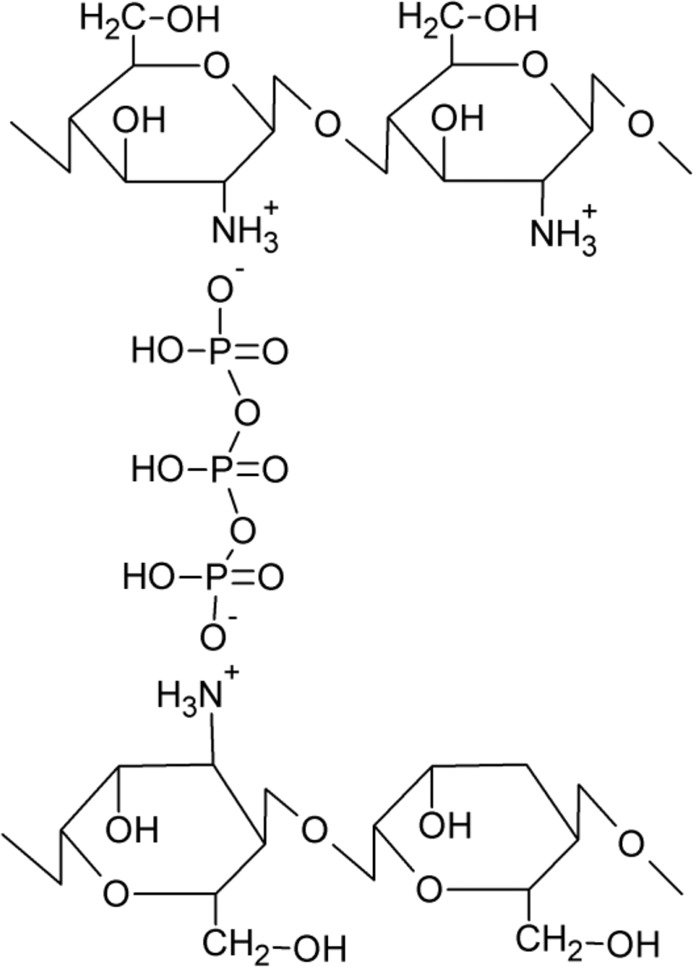
Example of ionically cross-linked chitosan [12]

Noteworthy characteristics that render chitosan suitable for biomedical and cosmetic applications cover minimal foreign body reactions, an intrinsic antibacterial nature, and an ability to be moulded in various geometries and forms such as porous structures suitable for cell ingrowth and osteoconduction. Due to its favorable gelling properties, chitosan can deliver morphogenic factors and pharmaceutical agents in a controlled fashion. Injectable hydrogels which can deliver cells and/or active compounds into a localized lesion site within any defect shape in a minimally invasive manner are mainly used in medicine, whereas for cosmetic applications hydrogels are used externally. [25, 26]. Covalent chitosan cross-linking leads to the formation of hydrogels characterised by a permanent network structure since irreversible chemical links are formed. This type of linkage allows water and/or bioactive compounds absorption without dissolution and permits the release of an active substance and/or drug by diffusion under pH-controlled conditions. Ionically cross-linked chitosan hydrogels exhibit a higher swelling sensitivity to pH changes than covalently cross-linked chitosan hydrogels. This fact extends the scope of potential applications of ionically cross-linked chitosan since dissolution can occur under extreme acidic or basic pH conditions. Chemical and ionic cross-links between chitosan molecules are shown in Figs 3 and 4. For hydrogels preparation, chitosan was cross-linked using genipin [27, 28], UV-irradiation [29–31], ionic cross-linking agents [32–34], chemical cross-linking agents [35–41]. Hydrogels have been prepared using blends of chitosan with other polymers and/or biopolymers [42–51]. Not only pure chitosan is used for hydrogels preparation. Hydrogels can be prepared based on chitosan derivatives, e.g. carboxymethyl chitosan [52–54]. PEGylated chitosan derivatives can also be used for hydrogel preparation [55].

The topical application of chitosan hydrogels in cosmetics is related to the film-forming and hair fixing properties of chitosan [56]. Chitosan hydrogels applied topically onto skin operate as humectants which are cosmetic preparations intended to increase water content in the top skin layers. Chitosan can also be combined with low molecular weight compounds, for example with pyrrolidone carboxylic acid (PCA) [57, 58]. Moisturizing products increase water content in skin and help keep it soft and smooth. For chitosan hydrogels, the moisturizing properties depend on the molecular weight and deacetylation degree of chitosan [59].

Chitosan has been included in a range of hair products, such as shampoos, rinses, permanent wave agents, hair colorants, styling lotions, hair sprays and hair tonics [60]. A chitosan gelling ability was also used in hydroalcoholic mixtures to formulate chitosan-based gel form for cosmetic applications [60, 61]. Chitosan can also be used in oral care cosmetics [62, 63]. Oral care is focused on preventive activity the aim of which is to avoid dental and gum illnesses. For this reason, the effect of chitosan hydrogels on oral biofilm formation is widely studied [64, 65]. In oral care, chitosan gels have also been used as a vehicle for other therapeutic products with different activities [66]. For such a purpose, blends of chitosan with other polymers can also be used. For example, chitosan-hydroxypropyl methylcellulose 3D hydrogels containing O-toluidine for antimicrobial photodynamic inactivation were produced and tested against *S. aureus*, *A. actinomycetemcomitans* and *P. gingivalis* biofilms. These hydrogels showed promising results regarding their clinical use with an appropriate delivery of o-toluidine [67].

## Hyaluronic acid

**Fig. 5:**
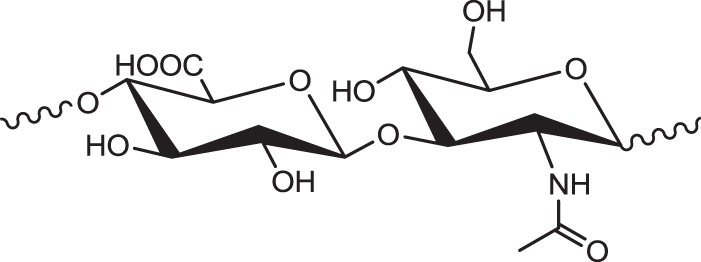
The structure of hyaluronic acid

Hyaluronic acid (HA) is a member of the glycosaminoglycan family - linear polysaccharides consisting of alternating units of *N* -acetyl-D-glucosamine and glucuronic acid which can be found in every tissue in vertebrates [68]. HA can be considered the largest glycosaminoglycan of molecular weights up to several millions. The HA structure is shown in Fig. 5. Unlike other members of the glycosaminoglycan family present in the human body (such as chondroitin sulfate, dermatan sulfate, keratin sulfate and heparin sulfate), HA is not covalently bonded to proteins. HA is water-soluble and forms highly viscous solutions with unique viscoelastic properties. HA can be arranged in three-dimensional structures in solutions with extensive intramolecular hydrogen bonding. The hyaluronic acid structure shows remarkable ability to entrap approximately 1000 times its weight of water. HA plays an important structural role in a variety of tissues including the articular cartilage, nucleus pulposus, skin, cervis, and glycocalyx of endothelial cells. This naturally-occurring biomolecule has commonly been used to inject into the dermis (as dermal filler) to restore skin volume and minimize the appearance of wrinkles as well as nasolabial folds. The unique properties of HA that mimics the natural materials found in our cells guarantee effectiveness and safety as well as tolerability to patients.

Due to its high functionality and charge density, HA can be cross-linked by a variety of physical and chemical methods. It also plays an important role in tissue repair by promoting mesenchymal and epithelial cell migration and differentiation, thereby enhancing collagen deposition and angiogenesis. This property, in addition to its immunoneutrality, makes HA an ideal biomaterial for tissue engineering, drug delivery and wound healing applications. Its aqueous solubility allows HA to be fabricated into different types of porous and three-dimensional structures for these applications. HA hydrogels properties depend on the initial concentration of a HA solution and on cross-linking agents [69]. The swelling ratio is also dependent on the cross-linking process. HA chains can be bonded with chemical cross-linkers [69–73]. A cross-linked HA hydrogel with a three dimensional network structure is more resistant towards enzymatic degradation than native HA due to the formation of bridges and intermolecular bonds between HA chains and the cross-linker. The hydrogel stiffness may be affected by HA viscoelasticity; a rheological characteristics depends also on HA concentration and chains cross-linking. HA hydrogels show promising results in skin rejuvenation and general skin appearance improvement [74]. HA has been used as dermal filler, in intradermal injection, scaffolds, creams, films, foams, and gels for treating different types of diseases [75–78]. HA hydrogels have also been used to treat wide ranges of skin problems including wrinkles, nasolabial folds, anti-aging, skin augmentation, skin hydration, and collagen stimulation. The promising anti-wrinkle potential of HA-based formulations was shown by several authors [79–81]. The anti-wrinkle efficacy of HA depends on its molecular weight due to differences in the percutaneous absorption of HA of different molecular weights across the stratum corneum [78]. HA hydrogels can also be used for hair treatment; however, since HA is costly, this biopolymer is rather rarely used in hair treatment cosmetics [82].

Nowadays, HA has become one of the most crucial ingredients in cosmetic as well as nutricosmetic products. Almost all products for mature skin that exhibit moisturizing, skin protective, and anti-aging properties contain HA. Its high ability to replenish skin moisture results in softer, smoother, and radiant skin. Skin hydration leads to slowing down the wrinkle formation and improves deep fine lines of already developed wrinkles which generally appear upon aging.

## Other biopolymers for hydrogel preparation

**Fig. 6:**
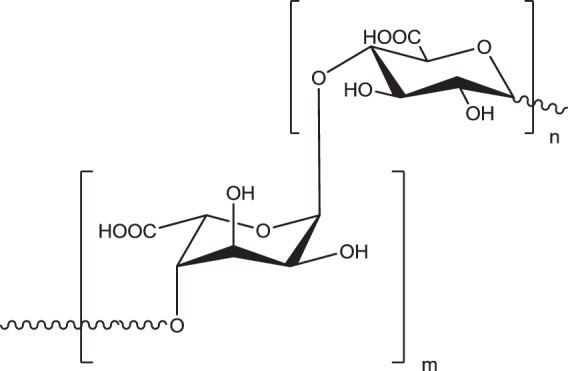
The structure of alginic acid

Several other biopolymers can be used for hydrogels preparation for cosmetic applications [83]. From among others, alginic acid and sodium alginate can be distinguished. Alginate is a non-branched, binary copolymer of (1-4) glycosidically linked β -d - mannuronic acid and α -l - guluronic acid monomers (Fig. 6). The alginate composition (ratio of the two uronic acids and their sequential arrangements) varies with a source of its extraction. Alginic acid is present within the cell walls and intercellular spaces of brown algae. It provides flexibility and strength to marine plants. Due to its non-toxicity, alginate has been extensively used as a food and cosmetic additive. Alginate hydrogels have been studied as alternative injectable materials to hyaluronic acid [84].

Carrageenan-based hydrogels can also be considered for cosmetic applications [85]. However, such hydrogels have also been used in food industry as gelling, stabilizing agents and thickeners because of their high hydrophilicity, mechanical strength, biocompatibility, and biodegradability [86].

Many natural polymers play a significant role in cosmetic formulation as thickening agents due to their gelling properties. For example, cellulose gum is a water-soluble polymer derived from cellulose by introducing carboxymethyl groups on the cellulose backbone. Such an anionic cellulose molecule hydrates and dissolves readily in water. Cellulose gums provide rheological control in thickening, suspension and emulsion stabilization as well as film formation, allowing the formulators to tailor the physical properties and performance of personal care products. Cellulose gums provide a thick but non-tacky feel. Especially nanocellulose hydrogels show a great promise in a wide range of biomedical, energy storage, construction, separation, cosmetic, and food applications [87, 88]. Hydrogels based on dextran can also be used for cosmetic applications [89, 90].

Not only polysaccharides but also proteins have been used for hydrogels preparation for potential cosmetic uses [91, 92]. One of them is silk fibroin produced by the domesticated *Bombyx mori* silkworm. It has been employed as a suture material for decades. Natural fibroin is comprised of repeating alanine and glycine sequences that readily form β-sheet crystals responsible for the mechanical properties of silk. Natural (and also synthetic) silk elasticity and strength make them an important candidate for applying in synthetic bones, ligament, and cartilage. Pure fibroin is biocompatible, can degrade slowly in vivo, supports attachment and proliferation of many cells and supports osteoblastic differentiation. Silk fibroin can be processed into films and scaffolds to improve tissue regeneration in skin, nerves, bones and cartilage. Silk fibroin hydrogels with excellent mechanical properties were generated via a binary-solvent-induced conformation transition strategy. New hydrogel systems showed potential applicability for biomedical and cosmetic uses [93]. In cosmetic formulation, silk is usually used in the hydrolyzed form as a component of hair conditioners, body creams and hair sprays.

Keratin is one of the most abundant proteins in the animal population. It is the major component of hair, feathers, nails and horns of mammals, reptiles, and birds. This fibrous protein is composed of several repeating sequences of amino acids along its chain. The sequence of amino acids defines the possibility of intermolecular links formation and the access of amino acids to the chemical reaction. The presence of cystine (amino acid containing sulfur) in the keratin chain leads to characteristic inter and intramolecular disulfide bonds which determine keratin properties. From the polymer science viewpoint, keratin is a biopolymer insoluble in water and common solvents. Soluble keratin and/or keratin hydrolysates can be easily blended in the same solvent with other polymers and then thin films and hydrogels can be prepared by the solvent evaporation. Such thin films can be used as a biomaterial as well as a material for covering other materials made of synthetic polymers. As a waste material, keratin is relatively inexpensive and its application in the medical field may reduce cost of the production of new biomedical materials. As keratin is the main hair component, it is mainly used for hair conditioning. Water soluble hydrolyzed keratin is a component of hair conditioners, body creams and hair sprays. The human hair-derived keratin-based in situ cross-linkable hydrogels that can serve as a dynamic matrix for enhanced wound healing were prepared [94]. It was demonstrated that the developed keratin-based hydrogels accelerate the wound healing process in a full-thickness animal. It can be assumed that hair-derived keratin-based hydrogels may also be potentially used in cosmetics.

In Table 1, a summary of biopolymers used for cosmetic purposes in hydrogel forms together with their advantages and disadvantages has been shown.

**Table 1:** Summary for biopolymers used in cosmetic applications in hydrogel form

S. No.	Biopolymers used as hydrogels in cosmetic applications	Advantages	Disadvantages
1	Kappa-carrageenan	• holds water onto skin and hair • used as a conditioning agent for hair • acts as a moisturizer	• may cause inflammation • may cause irritation
2	Xanthan gum	• withstands different temperature ranges and pH values • skin conditioning properties • non-toxic	• may cause irritation
3	Guar gum	• hair and skin condition is improved • prevents water loss • increases product self-life	• allergic sensitivity
5	Pectin	• strengthens skin	• pectin from a few sources may occasionally show poor gelling ability
6	Alginate	• erases fine wrinkles • increases elasticity and strengthens skin • makes skin look fresh	• gel formed may have foul smell
7	Cellulose	• increases the amount of moisture in skin • minimizes the appearance of hyperpigmentation	• shows poor compatibility with a hydrophobic polymer matrix
10	Gelatin	• improves skin health • causes skin firmness	• may cause allergic reactions
11	Collagen	• improves skin elasticity • reduces wrinkles • boosts skin hydration	• may cause local cutaneous necrosis and inflammation responses
12	Hyaluronic acid	• helps reduce the visibility of fine lines and wrinkles • leads to smoother skin	• may cause allergic reaction • may cause rash on the application site
13	Chitosan	• antimicrobial • antioxidant • softens the skin	• cross-linking of chitosan gel may affect chitosan intrinsic properties

## Biopolymer blends

Hydrogels can be prepared not only from a single biopolymer. According to trends in polymer and biopolymer science, a new way of hydrogel preparation is based on blending two or even more polymers and/or biopolymers [12, 95, 96]. There are several studies on the blends made of collagen and chitosan [97–103], Good swelling properties have been found for ternary blends made of collagen, chitosan, and hyaluronic acid [104–107].

Chitosan and polyvinyl alcohol blends [108] as well as poy(vinyl alcohol) and collagen-based hydrogels have also been proposed [109]. Chitosan–polyvinyl pyrrolidone hydrogels have been proposed mainly for biomedical applications; however, such hydrogels can also be applied in cosmetics [110–112]. Hydrogels made of polymer blends can be effectively used to deliver active cosmetic ingredients. A therapeutic hydrogel made of poly(vinyl alcohol) or poly(vinylpyrrolidone) applied in the atopic dermatitis treatment was proposed [113]. Such hydrogels can also contain a medicinal plant extract which could be used in dermatitis treatment. Poly(vinyl alcohol) itself together with its blends with other polymers can be used for preparing good quality hydrogels that constitute components of pharmaceutical, cosmetic and medical products [114]. Synthetic polymer hydrogels for biomedical applications are widely studied, but most of them can also be applied in cosmetics [115]. Because of a large number of publications regarding hydrogels based on synthetic polymers and their blends, this short review is focused mainly on hydrogels made of biopolymers with potential cosmetic applications.

## Bigels

Bigels are unique biphasic systems arranged by combining hydrogels and oil based organogels. In pharmaceutical and cosmetic products bigels are found due to their ability to deliver both hydrophilic and hydrophobic drugs [116]. Since bigels show features of both an oil (organogels) and water (hydrogels), they impart both the hydration and moisturizing effect to the stratum corneum of human skin. Simultaneously, they improve drug permeation to different skin layers on topical application [117]. Bigels can be prepared by mixing an organogel in a hydrogel and vice versa. However, hydrogel-in-organogel systems are less explored. Several biopolymers including sodium alginate, starch, carboxymethyl cellulose [118], guar gum [119], gelatin [120], etc. have been used as hydrogel components of bigel systems. Moreover, a low methoxy pectin hydrogel containing an olive oil-based organogel was prepared for pharmaceuticals and cosmetics delivery [117]. For the cosmetic formulation of bigels, it is very important to ensure the mechanical, textural properties, smoothness and thickness of bigels which can be studied by rheological measurements performed by changing organogel and/or hydrogel proportions. For example, different bigel formulations based on guar gum and sesame oil were prepared for the controlled ciprofloxacin release [119]. Bigel systems can be used to deliver drugs showing poor bioavailability when taken orally and thus dose frequency can be reduced. For instance, hydroxypropyl-methylcellulose hydrogel and sorbitan monostearate organogel were used to prepare a bigel system for transdermal delivery of a drug used in hypertension and angina treatment [121]. Bigels are expected to deliver various types of drugs and cosmetics; however, their physico-chemical properties should be well characterized before they find therapeutic applications.

## Hydrogels for active compounds encapsulation

Hydrogels based on biopolymers can compose materials especially suitable for active ingredients encapsulation in cosmetics and other biological elements [122]. Encapsulation has been widely researched for pharmaceutical, food, and cosmetic purposes. Hydrogels are especially useful for biological agents encapsulation because they provide the natural aqueous environment required for biomolecules to function in biological systems [123, 124].

Cosmetic products often contain biologically active substances that are unstable and sensitive to temperature, pH, light and oxidation. These substances may undergo undesirable reactions that lead to the reduction or even loss of their effectiveness. Such reactions may also result in cosmetic products degradation [125].

Encapsulation has been proposed not only to increase cosmetic products stability and protect them against degradation, but also to control the release of active ingredients used in cosmetic products [126]. Industrial and academic sectors are especially interested in cosmetics ingredients microencapsulation. There are several patents regarding microencapsulation in cosmetic and personal care products [127–130]. For cosmetic applications, hydrogels are ideal for the encapsulation of proteins, peptides, fragrances, antioxidants, sun filters, fragrances, moisturizers and anti-aging, tanning, and whitening agents [125]. The hydrogels properties required for encapsulation can be selected according to specific applications. They can be stimuli-responsive, degradable, highly or lightly cross-linked. Biopolymers for such hydrogels preparations can be functionalized with biological molecules in order to provide the required biochemical cues for the encapsulated molecules or cells (e.g. proteins and peptides encapsulation). For example, cholesterol-functionalized carbonates with a central hydrophilic block were shown to assemble into gels that could encapsulate drug-loaded polymeric micelles [131].

Most commonly used shell materials in cosmetics include polysaccharides (gums, starch, cellulose, cyclodextrines and chitosan), proteins (gelatin, casein and soy proteins), lipids (waxes, paraffin and oils) and synthetic polymers [acrylic polymers, polyvinyl alcohol and poly(vinylpyrrolidone)] [132–136]. Their mechanical properties and degradation rate are strongly defined by the structural characteristics and molecular weight of a polymer and nature of a chain end groups. To improve the properties of these polymers, chemical functionalization can be employed [137–140].

## Future perspectives of hydrogels for cosmetics

Hydrogels are widely used in biomedical fields. There are many papers published so far regarding hydrogel for controlled drug delivery, shape memory implantable devices, tissue engineering and regenerative medicine. However, when comparing the results and existing knowledge about hydrogels in cosmetics, the small amount of papers regarding the hydrogels in cosmetics constitute the major problem.

According to the Scopus Data Base, about 68,930 papers have been published in which the word «hydrogel» appears in a title, keyword, abstract (only in an article title - about 24,762). These data show that many research groups have been working with hydrogels so far. However, only about 20 papers discuss hydrogels in cosmetics when we use the word «hydrogel» and «cosmetic» in order to find results. The results concern only the combination of the keywords in titles. When the search takes abstracts into account, there are more results, about 464 documents. These data were collected in March 2020. Probably, there are also reports not provided by Scopus, so they are not easily accessible.

It is worth to mention that the selected hydrogels used in biomedical fields can be also suitable bioadhesive hydrogel formulations for cosmetic application on skin. In fact, biopolymers used for preparation of hydrogels for biomedical applications are similar to those used in biomedical applications (e.g. collagen, gelatin, hyaluronic acid, alginate, chitosan, cellulose and its derivatives).

Future perspectives of hydrogels for cosmetics can be similar to those for biomedical applications. So far in cosmetics have been used conventional hydrogels. The conventional hydrogels undergo only the swelling–deswelling process depending on the surrounding environment and availability of water. Recently, considerable interest has been drawn to the so-called ‘smart hydrogels’. Smart hydrogels have the ability to respond to changes in their external environment. Polymers for smart hydrogels can exhibit dramatic changes in their swelling behavior, sol-gel transition, network structure, permeability, or mechanical strength in response to changes in the pH, ionic strength, or temperature. In the skin and hair treatment several procedures have been used which are usually based on temperature, electric and magnetic fields, and light. These physical stimuli can be used for the stimuli-sensitive hydrogels in cosmetics. Apart from physical stimuli, also chemical stimuli including pH, ions, and specific molecular recognition events can be used in cosmetics. Some hydrogels can also respond to particular molecules like enzymes and sweat components, which may elicit a biological or biochemical response.

The area of hydrogels for cosmetic applications is neither a closed nor a completed topic. Future perspectives of such hydrogels will involve already existig hydrophilic polymers and biopolymers as well as polymer blends and derivatives.

## Conclusions

Numerous hydrogel structures have been prepared and characterized for cosmetic applications. In particular, bioadhesive hydrogels show one important advantage over conventional hydrogels as they allow longer residence time on the application site. The significant features of the hydrogel networks for applications in cosmetics include a swelling ability and mechanical strength. Biodegradability also constitutes an advantage of these materials. The structural modification capability of biopolymers may lead to the formation of new applicable derivatives. Active substances incorporation into a hydrogel structure as well as encapsulation using biopolymers as a shell can lead to new cosmetic products development. Hydrogel preparation, modified performance, and cross-linking mechanism are usually related to the appointed aim, for example preparation for hair, skin, nail, oral care. Active ingredients incorporation into a hydrogel structure exerts their action at deep skin layers. On the other hand, bigels offer a unique advantage of delivering both hydrophobic and hydrophilic drugs via the transdermal route. Biopolymers modification and/or blending of two or even more biopolymers can result in new hydrogels development for cosmetic applications.
